# Effects of Education Based on Health Belief Model on Dietary Behaviors of Iranian Pregnant Women

**DOI:** 10.5539/gjhs.v8n2p230

**Published:** 2015-06-25

**Authors:** M. Khoramabadi, M. Dolatian, S. Hajian, M. Zamanian, R. Taheripanah, Z. Sheikhan, Z. Mahmoodi, A. Seyedi-Moghadam

**Affiliations:** 1 Infertility and Reproductive Health Research Center, Shahid Beheshti University of Medical Sciences, Tehran, Iran; 2 Department of Reproductive Health and Midwifery, Faculty of Nursing and Midwifery, Shahid Beheshti University of Medical Sciences, Tehran, Iran; 3 Shahid Beheshti University of Medical Sciences, Tehran, Iran; 4 Department of Obstetric and Gynecology, Infertility and Reproductive Health Research Center. Shahid Beheshti University of Medical Sciences, Tehran, Iran; 5 Department of Reproductive Healths and Midwifery, Faculty of Nursing and Midwifery, Shahid Beheshti University of Medical Sciences, Tehran, Iran; 6 Department of Midwifery, Faculty of Nursing and Midwifery, Alborz University of Medical Sciences, Karaj Iran; 7 Department of cellular and molecular biology, Tonkabon branch, Islamic Azad University, Tonkabon, Iran

**Keywords:** prenatal education, health belief model, nutrition, health

## Abstract

**Introduction:**

Mothers and children are the most vulnerable members of every society. As a result many deaths occur in these two groups, so caring for these two groups is very important. Today, it is believed that the health of an infant is related to the health of their mother. Maintaining a healthy weight before pregnancy, and optimal weight gain during pregnancy by appropriate and sufficient nutrition, are two effective measures for the prevention of low birth weight.To provide successful health interventions, it is essential to design and implement effective health education programs. Successful education also depends on the proper use of theories and models in health education. The Health Belief Model is a model that illustrates the relationship between beliefs and health, and it is based on the hypothesis that preventive health behavior consists of personal beliefs. The aim of this study was to assess the effects of training on the Health Belief Model on dietary behaviors of a sample of pregnant Iranian women.

**Materials and Methods:**

This study was a randomized controlled clinical trial, involving 130 pregnant women who attended two health care centers of Shahid Beheshti University of Medical Sciences. Data was collected by a structured questionnaire in three parts and seven sub-scales (including demographic characteristics, knowledge and dietary behaviors) based on the Health Belief Model.

Principles of education were based on the Health Belief Model and performed twice during two-hour sessions in the intervention group. Women in the control group received routine care and did not receive training on the above model. In order to evaluate the intervention, the previously mentioned questionnaire was administered one month after completion of the intervention, and filled by participants in both groups. Data were analyzed by SPSS software and reported with diagrams and tables.

**Results:**

The mean score for each variable before the intervention, except for the performance guide variable, was not significantly different between the two groups (p<0.05).

A month after the intervention, the mean scores of the knowledge, perceived severity, perceived benefits in each group, were significantly different.

These results demonstrated that there were significant differences between the two groups in terms of mean scores of knowledge, perceived severity, perceived barriers, performance guide and individual performance, and the means of these variables in the intervention group were also higher than the control group. On the other hand, after the intervention, there was no statistically significant difference found in the mean scores of perceived benefits and perceived susceptibility between the two groups (two independent samples t-test, P <0/001).

**Conclusion:**

Educational interventions based on health promotion patterns can be effective in enhancing awareness, better understanding of risks, reducing barriers to healthy behavior and ultimately, improving women’s health and nutritional performance during pregnancy.

## 1. Introduction

Mothers and children are the most vulnerable members in any society, thus in the majority of countries, these two groups have higher mortality numbers.

A number of adverse pregnancy outcomes, including; intrauterine growth retardation, low birth weight, anemia, pre-eclampsia, preterm delivery and even fetal or infant death, are due to undernutrition or failure to receive all the necessary micronutrients (micronutrient deficiencies) during pregnancy (Bhutta et al., 2013).

Low birth weight, means a weight of less than 2,500 grams, and this produces an infant mortality rate factor in the region of 40-80%, with 98% of these mortalities occurring in developing countries (Abu-Saad & Fraser, 2010). Furthermore, some of the most important causes of maternal mortality, such as; pre-eclampsia, anemia and postpartum infections can be prevented by modifying the model of nutrition during pregnancy (Girard & Olude, 2012; Villar et al., 2003).

In addition, excessive weight gain during pregnancy is related to an increased incidence of complications, such as; preeclampsia, cesarean delivery, instrumental delivery^1^ and postpartum complications (Asbee et al., 2009). This problem has also been linked to childhood obesity, cardiovascular disease and Type 2 diabetes in adolescents (Dodd et al., 2014).

The World Health Organization (WHO) has introduced the obesity epidemic as one of the most significant threats to global health, particularly in women of childbearing age (Guelinckx, Devlieger, Beckers, & Vansant, 2008). Therefore, adequate weight gain via a balance between energy and protein intake, regular consumption of micronutrients or micronutrient rich foods is vital, since these decisions making processes both prior to becoming pregnant and during pregnancy play key roles in improving pregnancy outcomes (Ramakrishnan, Grant, Goldenberg, Zongrone, & Martorell, 2012).

Previous studies, particularly in low-income and middle-income countries, have shown that individual education and counseling during the first pregnancy visit helps mothers to improve their food consumption and the use of dietary supplements. This information should focus on the mothers’ needs and their fetus at three months of pregnancy and the need to maintain consistant weight gain in order to avoid long fasting or over-eating and its complications (Girard & Olude, 2012).

Poor weight gain, and more importantly, weight gain and obesity in mothers and infants during pregnancy results in subsequent complications and ongoing problems. Like many developing countries, there is a growing trend in these types of issues in Iran (Motlagh et al., 2011; Tabatabaei, 2011).

The results of several Iranian studies have suggested that calorie and protein intake is similar in both non-pregnant and pregnant women. As a result pregnant women are not receiving sufficient minerals or vitamins and they require nutritional supplements during pregnancy despite having a normal BMI or weight gain in pregnant women. This condition is often associated with mistakes found in the women’s nutritional habits and deficiencies in their nutritional knowledge, especially among middle and lower socio-economic groups (Delvarian Zadeh, Ebrahimi, & Haghighi, 2007; Eshghinia et al., 2014; Maddah, 2012; Mohammad Alizadeh Charandabi, Kamalifard, Ebrahimi mamaghani, Asghari, & F., 2012). The Health Based Model (HBM) is one of the best known frameworks that refer to the key role of people’s beliefs as a stimulus for behavior change implementation. Based on this model, when people understand the level of risk that an unhealthy behavior poses, and their susceptibility to the adverse consequences of their feelings, as well as understanding their behaviors, they become interested in methods to reduce their risks. By using learning methods to counteract and reduce existing barriers they are able to mitigate these adverse effects, moreover, they can change their attitudes and the range of positive behaviors will increase.

The model includes components of perceived susceptibility, perceived severity, perceived benefits, perceived barriers, and a performance guide (Gilmore, 2011; Martin, Haskard-Zolnierek, & DiMatteo, 2010).

This study assessed the knowledge, beliefs, perceptions and behaviors of expectant mothers with respect to prenatal nutrition. The study aimed to determine the effectiveness of educational interventions based on the HBM to improve the dietary behaviors during pregnancy in a sample of expectant mothers living in Tehran, Iran.

## 2. Material and Methods

### 2.1 Research Design and Setting

This study was a randomized controlled clinical trial, and 130 pregnant women participated from two therapeutic-health centers (Level I) Shahid Beheshti University of Medical Sciences in Tehran. Participants who met the following criteria were selected:

Pregnant women with low risk pregnancies, living in Tehran, their first single pregnancy, and pregnancy demands for couples.

All of the women first received a phone call and the purposes of research and education methods were explained to them. The women who were willing to participate in the study were included in the final list of subjects. The subjects were then randomized into two groups by a block randomization procedure and performed by Excel software.

### 2.2 Sampling

Written consent was obtained from all participants. The minimum sample size using power = 80 and was obtained in each group and a total of 130 patients were enrolled in the two groups (64 patients in each group).

### 2.3 Measurement Tool and Data Gethering

Data was collected by a structured questionnaire based on HBM (Table 1). The questionnaire was designed for the study’s objectives and consisted of 65 questions / phrases in three parts and seven sub-scales (including demographic characteristics, knowledge and dietary behaviors) based on the Health Belief Model. Training classes included two sessions of approximately two hour duration (10-12 am) (based on comfort and willingness of the participants), were held one week apart. Criteria considering healthy diet behaviors during pregnancy in the study’s questionnaire and the educational contents of classes were prepared based on National Health Services (NHS) advices and National Institute for Health and Care Excellence (NICE) guidance.

**Table 1:** Subscales in pregnancy knowledge based on the health belief model

Subscales	Phrase Examples
Knowledge	What is the minimal amount of fruits and vegetables that should be consumed during pregnancy? (How many fruits and how many vegetables)
Perceived susceptibility	Because I’ve never had nutritional problems during pregnancy, therefore it will not happen for me.
Perceived severity	Nutritional problems in pregnancy can cause dire consequences for mother and her baby.
Perceived benefits	Recommendations and presentations about nutrition during pregnancy will lead to a healthy baby.
Perceived barriers	Preparing the required foods during pregnancy should take a lot of time.
Cues to action	Family members and my husband are recommending that I pay attention to my nutrition during pregnancy.
Dietary behaviors	How much dairy and milk products do you consume?

All of the education sessions were conducted by the first researcher who had adequate experience in the field of health education and full understanding of this mode of delivery.

At the beginning of the classes, a question and answer session was conducted for the initial survey of the women’s knowledge, this was followed by a lecture, presentation of posters, photographs, and training pamphlets, and then continued with group discussions. All participants were followed up by the researcher, and a month later, both groups were re-evaluated by questionnaires. After final evaluation, mothers in the control group were given one day of training and received an educational pamphlet.

The correct answers were given a score of 1 and incorrect answers were given a score of 0.

In addition, the question components of the model had 6 point Likert score options that ranged from strongly disagree (score 0) to strongly agree (score 5).

Scoring of the questions or phrases for dietary behaviors were considered as well as ratings:

Level 1 (poor dietary behaviors scores of 0-4), participants who had poor dietary behaviors or did not folliw the healthy, level 2 (near to the appropriate dietary behaviors, scores of 5-8) who met the minimums of standards sometimes, and level 3 (optimal dietary behaviors, scores of 9-12), who acted strictly in accordance with the instructions for proper nutrition during pregnancy (Table 1).

### 2.4 Ethical Considerations

Ethical approval for the study was obtained from the ethical review group and the Deputy of Research at the Shahid Beheshti University of Medical Sciences. In addition, Informed consent was obtained from all study participants.

### 2.5 Statistical Analysis

Data were analyzed using SPSS software, version 20. Missing data and outliers were checked before data analysis. Data from 130 pregnant women who participated in this study were included in these analyses. Between-group and within-group differences were measured using the two -independent samples t- test and paired samples t- test for the data had a continuous spread, respectively and the chi-square test was used for categorical variables to test the association between the variables. Moreover, correlation between the model’s variables was calculated applying linear correlation and calculation of Pearson’s correlation coefficient. Statistical significance was considered at the 5% level.

## 3. Results

Overall, all 130 patients completed the study and the response rate was 100%. The mean age of the participants (4.71 ± SD) was 26.22 years, the majority (77%) were in their first pregnancy, 43% were in their second pregnancy, while the others had more than two pregnancies and live births.

The mean gestational age of the participants was 21 weeks (SD=0.88), in addition, the majority had attained secondary education (43%) and were housewives (92%). There were no significant differences between the intervention and the control groups in regard to their demographic characteristics, as shown in Table 2.

**Table 2:** Comparison of participants’ characteristics in study groups

Group			P.value	Statistical test result	df

Variable	experiment	control			
*Age* (Mean difference ±SD)	26 ± 4.04	26.53±5.03	N.S	t= -0.64^a^	129
*Educational level*					
0-6 years(primary)%	21.6%	13.8%	N.S	1.88^b^	2
7-12 years(secondary)%	56.9%	67.7%			
>12 years(colledge)%	21.5%	18.5%			
*Woman’s occupation*					
Housewife	90.8%	93.8%	N.S	2.57^b^	1
Employee	6.2%	9.2%			
*Occupation of spouse*					
Jobless	2%	1%	N.S	11.09^c^	1
Employee	98%	99%			
*Number of pregnancies*					
(Mean difference ±SD)	1.5 ± 0.71	1.5 ± 1.00	N.S	-0.10^a^	129
*Gestational age (week)*					
<20	30%	35.5%	N.S	3.02^b^	1
>20	60%	64.5%			

a . Two independent samples t-test

b . chi-square

c . fisher’s exact test

At first, all model constructs scores was compared applying two independent samples t- test in two experiment and control groups before the intervention. Results showed that there were no significant differences, except for perceived susceptibility, as showed significant differences in the intervention group after the intervention.

On the other hand, at the begining of the study, no significant differences were observed between the two groups in terms of dietary behaviors, and all participants were in level 1 or 2 as defined before during pregnancy; but after intervention, 86.7% of women in experiment and 13.3% of women in the control group, were in level 3 of dietary behaviors, which indicates that the majority of women who received educations based on the HBM, achieved optimal dietary behaviors and this was a statistically significant difference (P <0.001, x^2^).

In the second stage, after educational intervention, using two related samples t-test, significant differences were seen in all model’s constructs, except for perceived susceptibility in the experiment arm(P<0.001), but there was not obtained such findings for control. For instance, mean scores of perceived susceptibility, perceived barriers and dietary behaviors in control arm did not significantly differ in the second stage compared with the first stage. However, for constructs knowledge, perceived benefit, perceived severity and cues to action, findings suggested significant differences in the second stage (Table 3).

**Table 3:** Withingroups comparing the mean scores of the subscales of prenatal dietary knowledge and behavior assessment tool based on the health belief model

Group									

Variable		Experiment				Control			

		Mean±SD	P.value*	t	df	Mean±SD	P.value*	t	df
Knowledge	before after	9.21±3.8 18.09±2.85	<0.001	18.38	64	8.55±3.96 10.92±3.74	<0.05	5.71	64
perceived susceptibility	before after	22.96±6 24.73±2.11	0.13	2.58	64	24.27±3.73 23.46±3.70	0.18	1.62	64
Perceived severity	before after	18.70±3.33 33.06±.37	<0.001	-28.26	64	19.52±2.99 30.01±4.68	<0.001	-20.72	64
Perceived benefits	before After	10±3.59 39.53±3.64	<0.001	-44.29	64	10.89±3.48 38.41±5.13	<0.001	-29.28	64
Perceived barriers	before after	22.01±9.67 27.53±12.17	<0.001	-3.81	64	23.10±7.25 23.32±7.25	0.51	-0.67	64
Cues to action	before after	24.40±8.14 30.04±4.12	<0.001	-5.31	64	26.98±5.70 28.10±5.91	<0.05	-.3.75	64
Dietary behaviors	before after	4.43±1.77 8.56±2.33	<0.001	-13.18	64	4.60±1.95 5.23±2.22	0.15	2.68	64

* Paired samples t-test.

In the third stage, mean diffences of the model constructs were compared using two independent samples t-test between two arms of study to assess the efficacy of the study intervention. Results manifested thet there were satistically differences for all variables, except for the perceived bebefit. This finding shows that the the majority of the model constructs have significantly progressed in the experiment compared to the control group (Table 4).

**Table 4:** Between groups comparing the difference between subscale scores of prenatal nutritional assessment tool based on the health belief model

Group			P.value*	t	df

Variable	control Mean difference ±SD	experiment Mean difference ±SD			
Knowledge	9.53±4.18	1.70±2.40	<0.001	13.07	129
perceived susceptibility	1.80± 0.70	-0.8±4.03	<0.003	2.98	129
Perceived severity	14.35±4.09	10.49±4.08	<0.001	5.38	129
Perceived benefits	29.53±5.37	27.53± 7.52	0.082	1.75	129
Perceived barriers	5.43±11.20	0.21±2.57	<0.001	3.59	129
Cues to action	5.64±8.56	1.12±2.42	<0.001	4.09	129
Dietary behaviors	4.13±2.53	0.63±1.25	<0.001	10.00	129

* Independent Samples t-test.

The major obstacles for the healthy diet and dietay behaviors noted by the participants were the time and expense needed to be spent for preparing the necessary and nutritive foods, especially proteins; however, this barrier was decreased among women in experiment arm but in the control group the perceived barrier still remained as the most important obstacle (12% vs 33%, P <0.001).

The greatest perceived benefit in the second stage of the control group was related to the beneficial effects of optimal nutrition on infant’s health (78%). Whereas in the intervention group, improvements were shown in maternal and fetal outcomes (67%), maternal awareness in regard to family health (54%), and attempts to eliminate feeding problems (44%).

Finally, Pearson’s coefficient was calculated with the correlation between the subscales in the intervention group, and after the educational intervention, the mean score of the subscales had a direct and positive relationship with the mean score of the women’s nutritional performance.

Among the subscales, the mean score of knowledge had the highest correlation with the mean dietary behaviors score, which indicates that the more increased awareness, dietary behviors was improved. However, among the model’s subscales, there was only a weak linear relationship found with the mean performance score, and this was not statistically significant (Table 5).

**Table 5:** Correlation between the model’s constructs in regard of the dietary behaviors during pregnancy

	Knowledge	Perceived susceptibility	Perceived severity	Perceived benefits	Perceived barriers	Cues to action	Dietary behaviors
Knowledge	1						
perceived susceptibility	0.283 P=0.022*	1					
Perceived severity	0.168 p=0.180	0.304* p=0.014	1				
Perceived benefits	0.10 p=0.61	0.11 p=0.19	0.450** p<0.001	1			
Perceived barriers	0.313* p=0.012	0.171 p=0.16	0.278* p=0.026	0.128 p=0.134	1		
Cues to action	0.21 p=0.068	0.231 p=0.065	0.305* p=0.013	0.130 p=0.142	0.107 p=0.158	1	
Dietary behaviors	0.467** p<0.001	0.203 p=0.103	0.233 p=0.061	0.278* p=0.028	0.206 p=0.086	0.12 p=0.19	1

* P<0.05;

** P<0.01.

Moreover, a weak linear relationship was shown between knowledge and performance subscales in the control group, but such relationship was not found for the other variables.

Participants’ demographic and socioeconomic characteristics in the study, including age, gestational age(less than 20 weeks and more), number of pregnancies,women’s and their spouse’s education level and occupational status were identified as possible confounders in the association between educational intervention and dependent HBM outcomes. Then, multiple linear regressions were appyed to assess the effect of prenatal education based on HBM on the model’s constructs, controlling for above mentioned confounders. Independent variables were excluded from the model if they were insignificant above a two-sided p-value of 0.05 and included if they were statistically significant. However, none of them changed the beta-coefficients of other variables when excluded. Model fit was assessed by adjusted R-squared for linear regressions.

## 4. Discussion

This study included 130 pregnant women attending two public health centers in Tehran. The results demonstrated that pregnant women need nutritional prenatal care, and require sufficient and proper knowledge in this area, the second important area is to educate expectant mothers about the risks of complications caused by a failure to comply with correct nutritional recommendations and the severity of potential risks arising from a lack of good nutrition on both the mother’s and their unborn child’s health. Motivation is required to make behavior modifications to produce good nutritional performance, or to replace former bad habits with optimal nutritional behavior instead. The study results indicated that providing group consultations and training to pregnant women, based on known patterns of health promotion, is a better short-term and cost-effective method of increasing the target group’s knowledge and inducing behavior modification than conventional methods.

Comparing the mean knowledge scores between the two groups, showed that there were significant differences in both groups at different stages of the study, therefore, we suggest that when giving information by maternal and neonatal health care providers, the needs of the target group must be taken into consideration. Furthermore, answering the women’s questions directly also played a more effective role than conventional methods, such as pamphlets, booklets, or media education. This is because the purpose of education is not only to deliver information, but it also enables the audience to assess their own thoughts, beliefs, and behaviors, regarding the information and then to make their decision on the changes they will need to make. Accordingly, effective training should take into account the audience’s needs; while on the other hand, the groups are influenced by the training, resulting in sharing in a bilateral teaching-learning process.

Evaluation of common beliefs is a useful strategy for health care providers in face-to-face situations. If the educator determines that the audience has incorrect beliefs, then the aim should be to be determining the reasons for the fallacy and to help the client to overcome the problem through the provision of correct information (Glanz, Rimer, & Viswanath, 2008). In Kazaemi et al.’s study the face-to face educations based on the HBM to pregnant women evaluated as an effective way to a reduction of environmental tobacco smoke exposure compared with the women in the control group (Kazemi et al., 2011).

Promotion of nutritional knowledge in society through nutritional education would go a long way to reduce malnutrition and this could be considered a necessity for developing countries. Selecting a suitable model for health education is the first step toward planning effective health promotion programs (Pallatino, 2013).

Malnutrition can arise from eating too few or too many calories and result in different adverse effects on pregnancy outcomes. As a result, nutrition is one of the most important factors discussed in various studies emphasizing the importance of health promotion interventions in this era.

In the present study, the majority of participants held a number of incorrect beliefs and insufficient information about the mother’s energy needs. This misinformation can lead to excessive intake of calories and create obesity in pregnancy; while on the other hand, reducing the intake of necessary food groups, such as proteins and minerals that are found in fruits and vegetables can lead to malnutrition.

Guelinckx et al. (2008) reviewed seven experimental studies conducted on women from low-income areas and these showed that educational interventions in the form of group training on adjusting energy intake and including essential micronutrients in the daily diet of pregnant women were important and considerably reduced the incidence of overweight and obesity during pregnancy (6 studies). Furthermore, they found improved glycemic index, plasma glucose and insulin concentrations (4 studies), and perinatal outcomes, compared with the control group (2 studies) (Guelinckx et al., 2008).

Inspite of improvement in Knowledge, perceived benefits and severity, dietary behaviors did not significantly progressed among women in control group in the second investigation compared with the first time. This finding may explain the impact of the model based antenatal education to facilitate decreasing perceived barriers and improve the specific nutrition knowledge into the healthy practice acheivement. de Jersey et al. observed although pregnancy specific nutrition knowledge of a sample of Australian expectant mothers was good, knowledge regarding recommendations for healthy diet during pregnancy such as enough fruit and vegetable consumption was poor and less than one in ten women met important dietary recommendations. These researchers suggest that although knowledge played a key role for behaviour change, nutrition knowledge was independently associated with intake of fruit and vegetables by women and this knowledge inadequacy can not lead to ppropriate behaviour (de Jersey, Nicholson, Callaway, & Daniels, 2013).

However, the results indicated an increase in perceived benefits, rather than improvements in eating behavior modification in both groups after the intervention. As a result of improvement in the control group’s performance, no statistically significant difference was found in the second stage response in the control group.

Mothers in the control group mostly knew the importance of healthy nutrition for having a healthy baby, in addition they were also aware that healthy families were related to healthy mothers.

The findings for the perceived barriers subscale for the control group remained in effect too.

These results imply an increase in the mothers’ perception of the benefits of healthy dietary pattern modifications over time, and the women are likely to be more sensitive to the health of their fetus and infant. The importance of correct nutrition notwithstanding, it may also contribute to the prevention of obstetric complications, such as; severe bleeding leading to anemia, impaired wound healing and delayed recovery after childbirth.

In this study, the women participating in the intervention group were able to dismantle perceived barriers to a greater extent than the control group, therefore, these findings indicate the effectiveness of the trainings was focused on correcting unhealthy beliefs and insufficient information.

For example, in the material given to the mothers in the intervention group, introduced food groups containing proteins were discussed, so if they did not have access to meat protein, inexpensive foods with high protein levels could be used to replace them. Moreover, if they had a milk intolerance or allergy to certain dairy products they could use other foods containing calcium, or if they could not eat fruit, they could introduce a variety of vegetables containing minerals and vitamins using simple and easy to apply explanations.

Safavi et al. (2006) studied 4,368 urban and rural women from 11 different regions of Iran, and they found that 42.7% of pregnant women in the second half of their pregnancy had iron deficiency anemia. This particular problem was greater in under resourced provinces and in the country’s southern and rural areas, problems were also more prevalent and severe in multiparous women and in these rural areas than in urban regions (22.2 to 27.8% versus 17.1 to 21.6%) (Safavi et al., 2006).

One of the causes of anemia and iron deficiency in Iranian pregnant women may be inappropriate patterns of food intake that can lead to deficient levels and low absorption of iron. Furthermore, a significantly large proportion of pregnant women with iron deficiency anemia lived in low-income areas, and as a result of their lower per capita consumption of protein products, such as meat, eggs, and other essential micronutrients, such disorders were more prevalent in this group of women (Safavi et al., 2006).

Like other studies, this study had some limitations. As the present study was carried out on a small and not very economically advantaged population in urban area and we did not find any differences bwtween education level or occupation status and dietrary behaviors; while findings from two studies showed that higher maternal education level had been associated with greater health knowledge among women (Perumal et al., 2013; Zhao, Kulane, Gao, & Xu, 2009) Moreover, health information was mostly delivered to this group of women through health care providers or mass media programs, thus, the results cannot be generalized to higher educated women. Secondly, impact of the HBM based education was evaluated in a short period (one month) after completion of intervention and maintenance of behavior change needs longer follow up.

## 5. Conclusion

The results of this study showed that improper dietary behavior habits need to be replaced with correct eating behaviors and the application of health promotion models such as the HBM can be combined with usual pregnancy care practices in health care centers. This effective form of health care delivery should be taken into consideration by maternal and neonatal health authorities. This type of education is low cost and can prevent expensive pregnancy complications and adverse obstetric outcomes, and be an effective method of containing the exorbitant costs imposed on families and the health care system.
